# Access to public healthcare services for transgender people: experiences in Brazil’s capital*

**DOI:** 10.1590/1980-220X-REEUSP-2025-0273en

**Published:** 2026-05-22

**Authors:** Ester Mascarenhas Oliveira, Carle Porcino, Cleuma Sueli Santos Suto, Hellen Torres Coelho, Paula Bastos Antunes, Dejeane de Oliveira Silva, Paulo Alberto Moura Marques, Luciana Neves da Silva Bampi

**Affiliations:** 1 Universidade de Brasília, Programa de Pós-Graduação em Enfermagem, Brasília, DF, Brazil.; 2 Escola Bahiana de Medicina e Saúde Pública, Salvador, BA, Brazil.; 3 Universidade do Estado da Bahia, Campus VII, Senhor do Bomfim, BA, Brazil.; 4 Centro Universitário de Brasília, Brasília, DF, Brazil.; 5 Universidade Federal de Minas Gerais, Residência em Obstetrícia, Belo Horizonte, MG, Brazil.; 6 Universidade Estadual de Santa Cruz, Ilhéus, BA, Brazil.; 7 Universidade de Brasília, Faculdade de Ciências da Saúde, Programa de Pós-Graduação em Enfermagem, Brasília, DF, Brazil.

**Keywords:** Transgender Persons, Health Services Accessibility, Equity in Access to Health Services, Barriers to Access of Health Services, Unified Health System

## Abstract

**Objective:**

To analyze transgender people’s experiences in accessing public healthcare services in Brazil’s capital.

**Method:**

This qualitative study involved 19 transgender individuals linked to three healthcare services in Brasília. The data, collected through in-depth interviews, were lexically analyzed using descending hierarchical classification with the aid of the software *Interface de R pour les Analyses Multidimensionnelles de Textes et Questionnaires*.

**Results:**

Five thematic classes emerged, organized into three axes: “Experiences and disputes for recognition in public and institutional spaces”; “Structural barriers and itineraries for access to healthcare”; and “Mental healthcare and successful experiences in welcoming”.

**Conclusion:**

Access to healthcare for transgender people is marked by structural, attitudinal, and symbolic barriers that reinforce inequality and exclusion in care. The positive experiences identified point to ways to strengthen inclusive practices, reaffirming health as a right guaranteed by Brazilian law.

## INTRODUCTION

Transgender people, or trans people, constitute an umbrella term encompassing transsexuals and *travestis* (a culturally specific gender identity in Latin America), whose gender identity does not correspond to the sex assigned at birth. Cisgender people, in turn, identify with the sex assigned at birth^(1)^. This contrast exposes deep tensions, marked by exclusion and violence produced by cisnormativity, which denies trans people full citizenship^(2)^. Considering these dimensions is fundamental to understanding barriers that affect access^(3)^ and care flows in the Brazilian Unified Health System (SUS - *Sistema Único de Saúde*).

International studies demonstrate common barriers to healthcare access for transgender people, whether in the United States of America (USA)^(4)^ or in Europe^(1)^, ranging from discrimination and inappropriate use of names to a lack of qualified professionals. In Latin American^(5)^ and African^(6)^ countries, this is compounded by a lack of public policies and economic limitations, which prevent transgender people from accessing private healthcare services.

In Brazil, these barriers limit comprehensiveness of care^(3,7,8,9)^, contribute to social exclusion and the invisibility of demands of transgender people in the SUS^(10,11)^, and highlight the incipient nature of the Brazilian National Policy for Comprehensive Health of Lesbians, Gays, Bisexuals, *Travestis,* and Transsexuals (PNSI-LGBT - *Política Nacional de Saúde Integral de Lésbicas, Gays, Bissexuais, Travestis e Transexuais*). Although the PNSI-LGBT^(12)^ was established in 2011 as a normative milestone, it has not undergone systematic review or assessment processes since then. This contributes to the difficulties observed in its implementation. In daily service routines, these difficulties manifest as the persistence of cisnormative practices and the discontinuity of care flows, which directly impact the health and disease processes of transgender people. Thus, the care environment becomes a “non-place”, reinforcing the invisibility of this group in terms of access^(10,13)^, which has direct consequences for the health of these individuals.

Situations of institutional neglect coupled with rights violations expose the limits of the protection guaranteed by public policies^(14)^, perpetuating inequalities, prejudices, and stigmas^(11,15)^ and hindering the realization of SUS principles^(3)^. The creation of specialized clinics, such as in the Federal District (FD) and in Pernambuco, represented an advance in the policy consolidation^(13,16)^. However, the existence of these services has not ensured effective access to care, given persistent institutional barriers^(17)^, which perpetuate the shortcomings of the right to health^(18,19,20)^.

In this context, marked by advances and setbacks in policies, Brasília, Brazil’s capital, is a strategic location for understanding the tension between norms and practices. Research on the health of transgender people in Brazil is limited and affected by regional inequalities. In the FD, the few existing studies have focused on the role of social movements in the implementation of trans clinics^(13)^. In light of this situation, and with the goal of promoting equitable practices within the SUS, this study aims to address the following question: how do transgender individuals access public healthcare services in Brasília, Brazil’s capital? The study will analyze the experiences of transgender people accessing public healthcare services in Brasília.

## METHOD

### Study Design

This qualitative, exploratory study analyzes transgender people’s experiences accessing public healthcare services in Brazil’s capital. The qualitative approach captures the meanings and trajectories in participants’ narratives, enabling the phenomenon to be systematized^(21)^. To ensure the study’s methodological consistency and transparency, the COnsolidated criteria for REporting Qualitative research criteria were followed^(22)^.

### Site

The study was conducted in Brasília, Brazil’s capital, in public healthcare services that serve as entry points for transgender individuals seeking care. These services included a Basic Health Unit (BHU), which is recognized for its role in serving this population due to its strategic location, the State Health Department’s transgender outpatient clinic, and the Mental Health Outpatient Clinic at the *Hospital Universitário de Brasília* (HUB). These services were presented as benchmarks of care. However, although they are essential, they operate in isolation without being integrated into care pathways.

### Population, Selection Criteria and Sample Definition

The number of participants was determined during the data collection process by considering their profiles, accessibility, and sample saturation. Due to the subject matter and difficulty accessing the target audience, the snowball sampling technique was used. At the end of each interview, participants provided new contacts. Data collection was finalized in the sixth wave due to thematic saturation^(21)^.

This technique was adopted to include respondents outside of formal service flows, which broadens the scope of the research and reduces institutional selection biases. Additionally, this technique was employed to facilitate access and address the ethical sensitivities associated with researching the transgender population.

Twenty-five people were invited to participate in the investigation at the end of the process. Nineteen of them accepted, meeting the following inclusion criteria: self-identifying as transgender, being 18 years of age or older, and having current or previous experience using any of the SUS healthcare services referenced in this work. Having a clinical, cognitive, or emotional condition that prevented participation in the interview was defined as an exclusion criterion. No one was excluded from the research, but six chose not to participate.

### Data Collection

This phase took place between March and June of 2022. It involved applying a sociodemographic questionnaire and conducting in-depth interviews. The first author recorded and transcribed the interviews. The interviews lasted an average of 50 minutes and were conducted in private. The interview guide consisted of 19 questions and was developed by the first author and reviewed by the supervisors to ensure clarity and methodological consistency. No pilot test was conducted. There was no prior contact between the researcher and the interviewees, reducing potential biases.

The lead researcher, a cisgender woman and public health nurse with experience in qualitative research and a doctoral candidate in nursing, collected the data. The researcher’s position contributed to the successful conduct of the interviews, facilitating attentive listening and a deeper understanding of the narratives.

It was made clear during the interviews that no evaluative judgments would be made. After the interviews, the researcher took reflective notes to support monitoring the data collection process. These notes did not form part of the study’s analytical *corpus*. Participants were informed that the data collection was related to academic research and not linked to healthcare services^(22)^.

### Data Analysis and Processing

The collected demographic data were transcribed and processed descriptively. To this end, a Microsoft Excel spreadsheet was used to calculate the absolute and relative frequencies, enabling the profiling of the investigated individuals.

The interviews were transcribed in full in digital format, organized into a single text *corpus*, and processed using the *Interface de R pour les Analyses Multidimensionnelles de Textes et Questionnaires* software^(23)^. The *corpus* underwent Descending Hierarchical Classification (DHC), a process that groups textual segments based on the frequency and co-occurrence of terms to generate statistically significant lexical classes. *Corpus* utilization indices and the percentage of retention of elementary context units (ECUs) were calculated to ensure the robustness of the analysis. The classes were interpreted based on Creswell’s precepts^(24)^, which included rereading, coding, building categories, and articulating connections between empirical findings and literature.

This methodological approach was articulated within a critical framework of gender, sexualities, and necropolitics. It engaged with trans authors’ works and sought to challenge disputes over intelligibility that affect the experiences of trans people within the SUS.

### Ethical Aspects

The research project was approved by the Research Ethics Committees of the *Centro Universitário de Brasília* (Opinion 5,292,617) and the *Fundação de Ensino e Pesquisa em Ciências da Saúde* (Opinion 5,419,246). Data collection was carried out after participants signed the Informed Consent Form. To preserve anonymity, participants were identified by the abbreviation “P” (for participant), followed by an Arabic numeral. The analysis identified thematic axes, the results of which are presented below.

## RESULTS

The sample consisted of 19 transgender individuals, including seven trans women, two *travestis*, and ten trans men. Participants’ ages ranged from 18 to 40 years old, with an average age of 27. Most of them (n = 10) had completed higher education, and two held master’s degrees. Twelve of these individuals reported a monthly income of up to two minimum wages.

**Figure 1:**
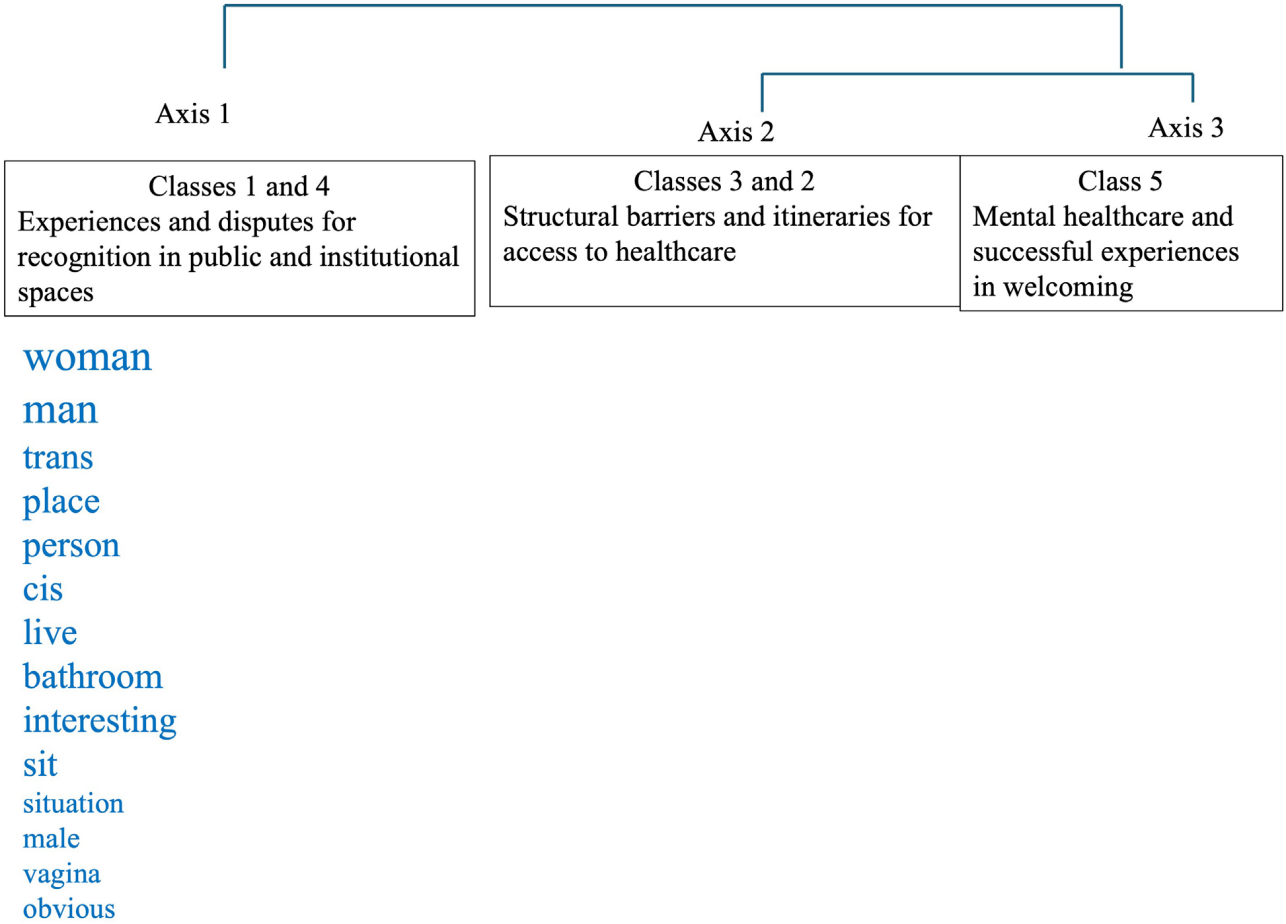
Dendrogram of the Descending Hierarchical Classification of interviews on access to healthcare services by transgender people in Brazil’s capital. Brasília, Federal District, 2022 (n = 19).

The *corpus* analysis yielded 19 texts, 1,511 segments, and 3,412 distinct forms with an average frequency of at least 12 per form. The DHC was applied to 75.58% of the corpus. Five classes emerged, highlighting different aspects of access to healthcare for transgender individuals. Class 1 accounted for 23.4% of the content, while the others had a relatively homogeneous distribution of ECUs. The discourses were analyzed along three thematic axes: axis 1 (classes 1 and 4) – Experiences and disputes for recognition in public and institutional spaces; axis 2 (classes 3 and 2) – Structural barriers and itineraries for access to healthcare; and axis 3 (class 5) – Mental healthcare and successful experiences in welcoming (Figure 1).

In axis 1, experiences related to recognizing gender identity as a prerequisite for healthcare access emerged. The narratives demonstrate that the invalidation of transgender identities within healthcare services causes embarrassment and restricts the right to health. This is evident in the way professionals treat patients, as one participant illustrated:

*I can’t be hypocritical; they don’t know how to respect gender identity.* (P2, trans woman, 22 years)

Discrimination and exclusion manifest beyond interactions with professionals in collective spaces such as healthcare services and public restrooms. These spaces should guarantee dignity and access, yet they often reinforce cisnormativity through statements such as:

*The doctor wouldn’t look at me; she asked my mother everything.* (P10, *travesti*, 35 years old)

*I felt very bad about some issues that came up regarding bathroom use.* (P14, trans man, 23 years)

These narratives suggest that barriers to healthcare access for transgender individuals stem from more than just the absence of services. These barriers range from the non-recognition of gender identities to the disconnect between established policies and their effective implementation.

Axis 2 emphasizes the daily challenges within the healthcare system, including delays in scheduling, repeated rescheduling, and a lack of resolution processes. Transgender individuals report resorting to various entry points without receiving adequate responses to their requests. This condition is evident in participants’ statements:

[…] *I first went to the health center, and they referred me to the Hospital de Base* […] *I received three negative responses from the endocrinologists, I tried three times* […] *the health center referred me to HMIB* (*Hospital Materno-Infantil de Brasília*) […] *she told me to go to the HUB* […]. (P1, trans man, 37 years)

There is frustration with the waiting and a devaluation of specific needs:

[…] *it basically takes many months or even years* […] *it is a waiting list that doesn’t know where it will lead you* […] *to get medication.* (P11, *travesti*, 33 years)

The interviewee’s perspective broadens our understanding of the barriers by highlighting the discrepancy between public policies and the reality of care. Although the right to health exists formally, it remains only on a normative level:

[…] *access to healthcare* […] *exists on paper, but it is not implemented. There are many trans people, travestis, from the interior. I’m from the interior of Minas Gerais; there is no policy for trans people there. I came to visit the trans clinic here* […] *I had to come to a capital city for reasons of improving my life. If I had stayed there, I would have been on my own hormone therapy, I didn’t have psychosocial support*. (P10, *travesti*, 35 years)

*There’s this thing called a general practitioner. A general practitioner won’t meet my needs. I need an endocrinologist.* (P6, trans man, 32 years)

Although access to healthcare for transgender people is formally guaranteed, some reports suggest that this right is undermined by denials and a lack of integrated services, which leads to inequality in care:

*When I received several negative responses, I gave up trying to get help through the SUS. I was wandering around the Federal District looking for a private doctor.* (P1, trans man, 35 years old)

*They* (BHU) *don’t respect gender identity; they tell you to go to the outpatient clinic.* (P7, trans woman, 28 years)

Axis 3 revealed practices that reduced constraints and promoted continuity of care. Participants reported experiences involving attentive listening, emotional support, and welcoming care, particularly within the realm of mental healthcare. The following statements illustrate these aspects:

*Psychologist, sometimes I think… this doctor of mine is amazing because he talks. Sometimes he doesn’t even say anything, he just observes me. Then he says something little* […] *it seems like him listening to me relieves and brings peace. I’ve never felt like this before.* (P3, trans man, 29 years)

[…] *I received psychological support from the Specialized Reference Center for Social Assistance for Diversity. I did, and the service was wonderful, I felt very welcomed and well assisted. But I also sought out the HUB. I also had psychological treatment there… the service was sensational*. (P8, trans woman, 25 years)

Participants reported situations in which they were treated respectfully and recognized by their chosen name:

*I was very well taken care of.* […] *the service is very good. I was respected. They called me by my chosen name from the beginning. I feel safe in this place.* (P1, trans man, 35 years)

[…] *wonderful! They know how to respect us; they ask if we want to undergo hormone therapy, because there are trans people who don’t want to take hormones, and then everything is done with a lot of care, love and respect, especially respect. They respect the chosen name above all. I feel very welcome.* (P2, trans woman, 22 years)

Support through referral services was mentioned, which highlights the importance of continuity of care and the inclusion of the family in the process:

[…] *I received a lot of psychological help at the referral center* […] *they did everything to make you feel welcome: they talked, asked about financial matters, if everything was alright, if you needed anything. I even had couples therapy with my wife. They referred me to the maternal and child hospital… I received excellent care from the psychiatrist.* (P5, trans man, 33 years)

In addition to mental health, there were positive comments about clinical practices. One interviewee mentioned the gynecological care provided, and another emphasized the importance of nursing:

[…] *I thought the gynecologist part would be much harder,* […] *the doctor gradually built a dialogue and explained the situation so that calmed me down,* […] *it was much easier than it could have been.* (P3, trans man, 29 years)

[…] *I needed to take the hormone. I went and the woman* (nurse) *treated me very well. She said, “Come here, buy the syringe and I will administer your medication”.* […] *at the Basic Health Unit as well.* (P5, trans man, 33 years)

Despite the challenges, this axis revealed positive experiences marked by respect, active listening, and effective clinical practices. These findings are grounded in the experiences of the participants and align with national and international literature, as discussed below.

## DISCUSSION

The reports presented in Axis 1 showed that discrimination and exclusion are not limited to the individual level and deny the legitimacy of gender identities. Participants reported a lack of respect for gender identity (P2) and embarrassment regarding the use of social and healthcare spaces (P14). Another study identified institutional denial expressed through embarrassment and a lack of welcome^(15)^.

Reports on P2, P10, and P14 indicate the presence of cisnormativity in healthcare services, including questioning of identities, denial of autonomy, and inappropriate use of intermediaries, such as family members. This pattern is not limited to one location. For instance, P10 reports being ignored by the physician. Cisnormativity naturalizes the binary gender norm, rendering certain forms of existence intelligible. This process creates rigid boundaries. Thus, when trans identities disrupt this logic, they are marginalized^(2)^. This is a system that reinforces prejudice and affects access to healthcare.

Studies in different countries show that prejudice continues to shape the lives of transgender people. Studies in the USA show that transgender individuals also face discrimination and social exclusion, which influences their access to healthcare. In Brazil, the literature reports similar barriers in care^(10)^, reinforcing that the barrier is structural. The reality observed in Brasília aligns with this scenario, in which cisnormativity permeates both the entry of transgender individuals into services and their ability to remain within them^(1)^.

The narratives of P10 and P14 demonstrate the delegitimization of identity and the denial of autonomy. These practices restrict the legitimacy of certain existences and perpetuate exclusions that affect access to healthcare. A study of 307 transgender individuals in Europe found that over 40% needed to convince healthcare professionals of the legitimacy of their needs^(4)^. This scenario reflects the difficulty of recognizing diversity, as well as the persistent pursuit of citizenship and the right to exist^(7,20)^. The social and institutional marginalization experienced by transgender individuals stems from historical inequalities^(14)^, underscoring the need for intersectoral collaboration to implement equitable public policies^(12)^.

Reports from axis 2 expose the concrete barriers that trans individuals face when accessing healthcare services, ranging from Primary Health Care (PHC) to specialized services. P1 describes long journeys and multiple refusals, and P11 highlights delays in obtaining medication, illustrating the “pilgrimage” individuals undertake in search of care. These findings suggest obstacles in recognizing and organizing care flows that are recurrent across different contexts within the SUS.

P10 highlights the need to travel in search of healthcare, which often leads transgender individuals to seek structured services elsewhere. This reflects a broader logic of exclusion. In practice, this means retracing steps and investing time and energy to secure basic rights. When healthcare services fail to respect chosen names and identity recognition, they create distance and force individuals to seek alternatives outside the SUS^(11)^.

In Belgium, the Netherlands, and Germany, structural barriers—such as service centralization and high transportation costs—affect care and well-being among trans individuals^(4)^. Delays in care due to structural and financial issues have also been identified in the USA^(1)^, showing that barriers to access and exclusion are not limited to Brazil but also occur in developed countries with greater technological resources. P10 also associates access to healthcare with the implementation of inclusive policies and services. However, such policies still show gaps in implementation, especially regarding funding, increasing inequalities in healthcare for transgender populations^(7,11,20)^.

As reported by P11, delays in scheduling and as described by P1, multiple referrals reveal the burden of logistical barriers. These obstacles directly impact care. Often, care is based on the individual initiatives of some professionals, without structured planning or outcome monitoring. In many cases, the search for body modifications occurs in contexts of vulnerability and invisibility^(9)^, which highlights how precarious conditions can shape personal choices and bodies.

The lack of specialists was highlighted by P6, “I need an endocrinologist; a general practitioner does not meet my needs”, indicating that academic training does not meet specific demands. In this context, prejudice^(25)^ and social stigma also emerge, negatively impacting healthcare for trans individuals^(7)^. Therefore, the political, structural, and institutional dimensions that shape access to healthcare for trans individuals within the SUS require critical review and effective transformation^(26)^. In practice, this means that some individuals progress within the SUS, while others face greater challenges, resulting in selective exclusion that determines which lives are recognized^(27)^.

The study data reveal an ongoing pursuit of care, which underscores the active and resilient nature of the transgender population despite barriers hindering access and exposing limitations in services. Despite this, resistance, exhaustion, and withdrawal also emerge^(20,25)^. Some participants reported alternative strategies: of the 19 participants, at least five mentioned self-medication, and six reported seeking private healthcare services. P1’s experience highlights institutional neglect. These narratives can be understood as forms of agency and resistance in which transgender individuals adapt their healthcare plans due to the lack of public services.

P7 mentions referral from the PHC unit, “They tell us to look for the outpatient clinic”. Thus, although the PHC unit is intended to be the gateway, it does not fully assume this role in addressing trans demands. This reflects selective care, with unequal effects on who receives care^(27)^. Studies in the USA^(28)^, Spain^(29)^, and Africa^(6)^ show that structural barriers are global.

The lack of preparedness of healthcare teams weakens the recognition of the needs of transgender people. This finding aligns with international studies that point to the difficulty of the PHC unit in acting as an effective entry point for transgender people into different healthcare systems^(27)^. Strengthening the PHC unit is strategic to overcome barriers and ensure comprehensive care through continuous professional training^(12,16)^. This disparity is also evident in Brasília, where only one specialized outpatient clinic exists, which is insufficient for demand^(13)^. In this context, the universalist proposal guiding the SUS faces significant implementation challenges^(26)^.

The findings indicate deficiencies centered on the PHC unit and a fragmented network that transfers responsibility for negotiating access to individuals. This scenario highlights the need for network integration to reduce inequalities and ensure continuity of care^(7,9)^.

Reports from axis 3 bring a dimension of welcoming care, expressed through the use of chosen names and attentive listening. P8 highlighted psychological care. P3 stated, “The gynecology approach made me feel more comfortable”. P5 added, “I even had follow-up as a couple with my wife”. These narratives reveal that, despite the barriers, sensitive practices exist, centered on listening, respect for chosen names, and adaptation to individuality. They also underscore the significance of family context and the acknowledgment of personal choices, reinforcing the importance of inclusive strategies within the SUS. These findings support studies that emphasize the role of family members in strengthening gender identity and safeguarding transgender people’s mental health^(4)^.

Psychological support is essential for a person’s well-being, as P8 mentioned, demonstrating the power of sensitive practices even amidst institutional scarcity. This data shows that the mental health of transgender people is not disconnected from the structural conditions that permeate access to healthcare. In this context, P5 emphasized the strategic role that nurses’ guidance plays in implementing inclusive practices, such as active listening and welcoming care, in accordance with equity guidelines^(30)^. Humanization practices strengthen bonds and reduce the impacts of discrimination/exclusion, reaffirming the importance of training healthcare teams^(11,17,19,20)^. Although these practices are still limited, they sustain trust and continuity of care, showing that the SUS can be a space of safety and recognition for transgender individuals.

The limitations of this study are related to the small number of participants and the specific SUS services in a limited urban context. These limitations prevent the generalization of the findings. This situated analysis expresses experiences and meanings produced in a specific territory and care arrangement. It recognizes that distinct institutional and regional contexts can produce different experiences of access and care for transgender people. Nevertheless, the results offer valuable insights into access and care dynamics by shedding light on institutional barriers, cisnormative practices, and organizational structures that impact care production. While this knowledge does not aim to be universal, it can support critical reflections and improvements to flows, practices, and care pathways in similar contexts.

## CONCLUSION

This study examined the experiences of transgender individuals seeking public healthcare services in Brazil’s capital. Although access is legally guaranteed, the results show that it remains conditioned by structural barriers and practices that reproduce institutional cisnormativity. The study revealed a coexistence of exclusion and welcoming experiences, emphasizing that alongside invisibility and symbolic violence, positive healthcare access practices also emerged, particularly in specialized care settings.

Reports showed that care was perceived as safe when guided by respect for chosen names and recognition of gender identity. This highlights the importance of equity as a principle of the SUS. Strategies to reduce barriers to healthcare include the decentralization of specialized services through flows between levels of care, improved welcoming care, and guaranteed ongoing intersectoral training. These strategies pave the way for truly emancipatory care for the trans community.

## Data Availability

The entire dataset supporting the results of this study is available upon request to the corresponding author.
