# Supplementary material for: Access to public healthcare services for transgender people: experiences in Brazil’s capital
Source: Rev Esc Enferm USP. 2026 May 22;60:e20250273. doi: 10.1590/1980-220X-REEUSP-2025-0273en (PMC13267785; doi:10.1590/1980-220X-REEUSP-2025-0273en)
Supplement: Translation

# Translation: Acesso aos serviços de saúde pública por pessoas transgêneras: experiências na capital do Brasil*

**DOI:** 10.1590/1980-220X-REEUSP-2025-0273pt

**Authors:** Ester Mascarenhas Oliveira, Carle Porcino, Cleuma Sueli Santos Suto, Hellen Torres Coelho, Paula Bastos Antunes, Dejeane de Oliveira Silva, Paulo Alberto Moura Marques, Luciana Neves da Silva Bampi

**Affiliations:** 1 Universidade de Brasília, Programa de Pós-Graduação em Enfermagem, Brasília, DF, Brasil.; 2 Escola Bahiana de Medicina e Saúde Pública, Salvador, BA, Brasil.; 3 Universidade do Estado da Bahia, Campus VII, Senhor do Bomfim, BA, Brasil.; 4 Centro Universitário de Brasília, Brasília, DF, Brasil.; 5 Universidade Federal de Minas Gerais, Residência em Obstetrícia, Belo Horizonte, MG, Brasil.; 6 Universidade Estadual de Santa Cruz, Ilhéus, BA, Brasil.; 7 Universidade de Brasília, Faculdade de Ciências da Saúde, Programa de Pós-Graduação em Enfermagem, Brasília, DF, Brasil.

**Keywords:** Pessoas Transgênero, Acesso aos Serviços de Saúde, Equidade no Acesso aos Serviços de Saúde, Barreiras ao Acesso aos Cuidados de Saúde, Sistema Único de Saúde

## Abstract

**Objetivo:**

Analisar as experiências de acesso aos serviços públicos de saúde por pessoas transgêneras na capital do Brasil.

**Método:**

Estudo qualitativo, que envolveu 19 pessoas transgêneras vinculadas a três serviços de saúde de Brasília. Os dados coletados por meio de entrevistas em profundidade foram analisados lexicalmente através da Classificação Hierárquica Descendente, com auxílio do *software Interface de R pour les Analyses Multidimensionnelles de Textes et Questionnaires*.

**Resultados:**

Emergiram cinco classes temáticas, organizadas em três eixos: “Vivências e disputas por reconhecimento nos espaços públicos e institucionais”; “Barreiras estruturais e itinerários para o acesso à saúde”; e “Cuidado em saúde mental e experiências exitosas no acolhimento”.

**Conclusão:**

O acesso à saúde por pessoas transgêneras está marcado por barreiras estruturais, atitudinais e simbólicas, que reforçam o processo de desigualdade e exclusão no cuidado. As experiências positivas identificadas sinalizam caminhos para fortalecer práticas inclusivas, reafirmando a saúde como um direito assegurado pela legislação brasileira.

## INTRODUÇÃO

Pessoas transgêneras ou pessoas trans constituem uma expressão guarda-chuva que abarca transexuais e travestis, cuja identidade de gênero não corresponde ao sexo atribuído ao nascer. Pessoas cisgêneras, por sua vez, identificam-se com o sexo designado ao nascimento^(1)^. Esse contraste expõe tensionamentos profundos, marcados pela exclusão e pela violência produzidas pela cisnormatividade, que nega às pessoas trans a plena cidadania^(2)^. Considerar essas dimensões é fundamental para compreender barreiras que afetam o acesso^(3)^ e os fluxos assistenciais no Sistema Único de Saúde (SUS).

Estudos internacionais demonstram barreiras comuns no acesso à saúde por pessoas trans, seja nos Estados Unidos da América (EUA)^(4)^ ou na Europa^(1)^, envolvendo desde a discriminação e o uso inadequado de nomes até a falta de profissionais capacitados. Em países da América Latina^(5)^ e da África^(6)^, somam-se a isso a falta de políticas públicas e as limitações econômicas, que impedem as pessoas transgêneras de acessarem serviços privados de saúde.

No Brasil, essas barreiras limitam a integralidade do cuidado^(3,7,8,9)^, contribuem para a exclusão social e a invisibilidade das demandas de pessoas transgêneras no SUS^(10,11)^, e evidenciam a incipiência da Política Nacional de Saúde Integral de Lésbicas, Gays, Bissexuais, Travestis e Transexuais (PNSI-LGBT). Instituída em 2011, a PNSI-LGBT^(12)^, embora represente um marco normativo, permanece sem processos sistemáticos de revisão ou avaliação desde então, o que contribui para as dificuldades observadas em sua implementação. No cotidiano dos serviços, essas dificuldades se expressam por meio da persistência de práticas cisnormativas e da descontinuidade dos fluxos assistenciais, repercutindo diretamente no processo de saúde-doença de pessoas transgêneras. Assim, o ambiente de cuidado se torna um “não lugar”, reforçando a invisibilidade desse coletivo no que se refere ao acesso^(10,13)^, com consequências diretas sobre a saúde dessas pessoas.

As situações de negligência institucional, somadas a um cenário de vulneração de direitos, expõem os limites da proteção garantida por políticas públicas^(14)^, perpetuando desigualdades, preconceitos e estigmas^(11,15)^, e fragilizando a concretização dos princípios do SUS^(3)^. A criação de ambulatórios especializados, como no Distrito Federal (DF) e em Pernambuco, representou um avanço na consolidação da política^(13,16)^. Contudo, a existência desses serviços não tem assegurado o acesso efetivo ao cuidado, diante de barreiras institucionais persistentes^(17)^, o que mantém fragilidades no exercício do direito à saúde^(18,19,20)^.

Nesse contexto, marcado por avanços e retrocessos nas políticas, Brasília, capital do Brasil, é um campo estratégico para compreender tensões entre norma e prática. A produção sobre saúde de pessoas transgêneras no Brasil é limitada e atravessada por desigualdades regionais. No DF, os poucos estudos existentes têm se dedicado a compreender a atuação dos movimentos sociais e a implementação do ambulatório trans^(13)^. Diante desse cenário e com o objetivo de subsidiar práticas mais equitativas no SUS, este estudo busca responder à seguinte questão: como as pessoas transgêneras experienciam o acesso aos serviços públicos de saúde em Brasília, capital do Brasil? Este estudo busca analisar as experiências de acesso aos serviços públicos de saúde por pessoas transgêneras na capital do Brasil.

## MÉTODO

### Tipo de Estudo

Trata-se de estudo qualitativo e exploratório, que analisa as experiências de acesso aos serviços públicos de saúde por pessoas transgêneras na capital do Brasil. A abordagem qualitativa permite captar sentidos e trajetórias a partir das narrativas das pessoas participantes, possibilitando a sistematização do fenômeno estudado^(21)^. Para garantir a consistência metodológica e a transparência do estudo, foram observados os critérios estabelecidos na lista de verificação *COnsolidated criteria for REporting Qualitative research* ^(22)^.

### Local

O estudo foi realizado em Brasília, capital do Brasil, em serviços públicos de saúde que atuam como porta de entrada para o atendimento de pessoas trans, incluindo uma Unidade Básica de Saúde (UBS), reconhecida pela relevância no atendimento desse coletivo em razão da sua localização estratégica, o ambulatório trans da Secretaria de Estado de Saúde e o Ambulatório de Saúde Mental do Hospital Universitário de Brasília (HUB). Esses serviços foram apresentados como marcos de referência assistencial. Embora sejam essenciais, atuam de forma isolada, sem integração em linhas de cuidado.

### População, Critérios de Seleção e Definição da Amostra

O número de participantes foi definido no processo de coleta de dados, considerando o perfil, o acesso às pessoas e a saturação da amostra. Utilizou-se a técnica de amostragem *snowball* adequada à temática e ao difícil acesso ao público investigado. Ao final de cada entrevista, as pessoas participantes indicaram novos contatos. A coleta de dados foi finalizada na sexta onda, em razão da saturação temática^(21)^.

Essa técnica foi adotada por possibilitar a inclusão de depoentes fora dos fluxos formais de atendimento, ampliando o alcance da pesquisa e reduzindo vieses institucionais de seleção. Ademais, a técnica foi utilizada por facilitar o acesso e pela sensibilidade ética que envolve as pesquisas com a população transgênera.

Ao final do processo, 25 pessoas foram convidadas a participar da investigação. Dessas, 19 aceitaram, atendendo aos seguintes critérios de inclusão: se autodeterminar pessoa transgênera; ter 18 anos ou mais; e possuir experiência atual ou prévia de utilização de algum serviço de saúde do SUS referenciado neste trabalho. A presença de condição clínica, cognitiva e emocional que impedisse a participação na entrevista foi definida como critério de exclusão. Nenhuma pessoa foi excluída da pesquisa, mas seis optaram por não fazer parte do estudo.

### Coleta de Dados

Esta etapa ocorreu entre março e junho de 2022, com a aplicação de questionário sociodemográfico e a realização de entrevistas em profundidade, gravadas e transcritas pela primeira autora. As entrevistas, com duração média de 50 minutos, foram realizadas em ambiente reservado. O roteiro de entrevista, composto por 19 questões, foi elaborado pela primeira autora e revisado pelas orientadoras, com o intuito de assegurar clareza e consistência metodológica. Não foi realizado teste piloto. Não houve contato prévio entre a pesquisadora e os depoentes, o que reduziu potenciais vieses de relação.

Os dados foram coletados pela pesquisadora responsável, mulher cisgênera, enfermeira sanitarista e doutoranda em enfermagem com experiência em pesquisa qualitativa. A posição da pesquisadora no campo contribuiu para a condução das entrevistas, favorecendo a escuta atenta e o aprofundamento das narrativas.

Foi explicitado que não havia juízo avaliativo durante as entrevistas. A pesquisadora realizou anotações reflexivas após as entrevistas, utilizadas como apoio ao acompanhamento do processo de coleta de dados, não compondo o *corpus* analítico do estudo. As pessoas participantes foram informadas de que a coleta de dados estava relacionada a uma pesquisa acadêmica, não vinculada à assistência prestada nos serviços de saúde^(22)^.

### Análise e Tratamento dos Dados

Os dados demográficos coletados foram transcritos e tratados de forma descritiva. Para esse fim, utilizou-se uma planilha do *Microsoft Excel*, obtendo-se a frequência absoluta e relativa, o que permitiu traçar o perfil das pessoas investigadas.

As entrevistas foram transcritas integralmente em formato digital, organizadas em um *corpus* textual único e processadas pelo *software Interface de R pour les Analyses Multidimensionnelles de Textes et Questionnaires* ^(23)^. O *corpus* foi submetido à Classificação Hierárquica Descendente (CHD), que agrupa os segmentos textuais a partir da frequência e coocorrência de termos, gerando classes lexicais estatisticamente significativas. Foram calculados índices de aproveitamento do *corpus* e percentuais de retenção das unidades de contexto elementar (UCEs), o que assegurou a robustez da análise. A interpretação das classes seguiu os preceitos de Creswell^(24)^, a partir das etapas que incluíram releitura, codificação, construção de categorias e articulação entre achados empíricos e literatura.

Esse percurso metodológico foi articulado a um referencial crítico de gênero, sexualidade e necropolítica, em diálogo com produções de autoras e autores trans, buscando tensionar as disputas de inteligibilidade que marcam a experiência das pessoas trans no SUS.

### Aspectos Éticos

O projeto de pesquisa foi aprovado pelos Comitês de Ética em Pesquisa do Centro Universitário de Brasília, sob Parecer nº 5.292.617, e da Fundação de Ensino e Pesquisa em Ciências da Saúde do DF, sob Parecer nº 5.419.246. Após a assinatura do Termo de Consentimento Livre e Esclarecido, a coleta de dados foi realizada. Para preservar o anonimato, as(os) participantes foram identificadas(os) com a abreviação da palavra participante (P) seguida por um algarismo arábico. A análise possibilitou a identificação de eixos temáticos, cujos resultados são apresentados a seguir.

## RESULTADOS

A amostra foi composta por 19 pessoas transgêneras, sendo sete mulheres trans, duas travestis e dez homens trans, entre 18–40 anos, com média de idade de 27 anos. A maioria (n = 10) havia completado o ensino superior, sendo que dois possuíam o título de mestre. Desses(as), 12 relataram renda mensal de até dois salários mínimos.

A análise do *corpus* resultou em 19 textos, 1.511 segmentos de texto, 3.412 formas distintas e frequência média ≥ 12 por forma. A CHD incidiu sobre 75,58% do *corpus*. Emergiram cinco classes, as quais evidenciaram dimensões do acesso ao cuidado em saúde por pessoas transgêneras. A classe 1 concentrou 23,4% do conteúdo, enquanto as demais apresentaram a distribuição relativamente homogênea quanto à quantidade de UCEs. Os discursos foram analisados em três eixos temáticos: eixo 1 (classes 1 e 4) - Vivências e disputas por reconhecimento nos espaços públicos e institucionais; eixo 2 (classes 3 e 2) - Barreiras estruturais e itinerários para o acesso à saúde; e eixo 3 (classe 5) - Cuidado em saúde mental e experiências exitosas de acolhimento (Figura 1).

**Figura 1:** Dendrograma da Classificação Hierárquica Descendente de entrevistas sobre acesso aos serviços de saúde por pessoas transgêneras na capital do Brasil. Brasília, Distrito Federal, 2022 (n = 19).

No eixo 1, emergiram experiências relacionadas ao reconhecimento da identidade de gênero como condição de acesso à saúde. As narrativas mostram que a não validação das identidades trans nos serviços gera constrangimento e limita o direito à saúde. Isso se expressa no modo como as(os) profissionais tratam a(o) usuária(o), como ilustra uma participante:

*Eu não posso ser hipócrita; não sabem respeitar a identidade de gênero.* (P2, mulher trans, 22 anos)

Além da interação com profissionais, a discriminação e a exclusão também se manifestam em espaços coletivos, como serviços de saúde e banheiros públicos. Esses espaços deveriam garantir dignidade e acesso, mas acabam reforçando a cisnormatividade por meio de verbalizações como:

*A médica não olhava para mim; perguntava tudo para minha mãe.* (P10, travesti, 35 anos)

*Me senti muito mal com umas questões que aconteceram em relação ao uso do banheiro.* (P14, homem trans, 23 anos)

Essas narrativas apontam que as barreiras ao acesso à saúde por pessoas transgêneras não se resumem à ausência de serviços, mas se expressam desde o não reconhecimento das identidades de gênero até o distanciamento entre políticas instituídas e a sua efetiva implementação.

O eixo 2 evidencia as dificuldades cotidianas no serviço, marcadas por morosidade nos agendamentos, sucessivas remarcações e ausência de fluxos resolutivos. As pessoas transgêneras informam recorrer a diferentes portas de entrada, sem receber respostas efetivas às suas demandas. Essa condição é expressa na fala da pessoa participante:

[…] *antes procurei o posto de saúde, e o posto me encaminhou pro Hospital de Base* […] *tive três negativas dos endócrinos, tentei três vezes* […] *o posto me encaminhou pro HMIB* (Hospital Materno-Infantil de Brasília) […] *ela me disse pra ir pro HUB* […]. (P1, homem trans, 37 anos)

Há frustração com a espera e a desvalorização de necessidades específicas:

[…] *demora basicamente muitos meses ou até anos* […] é *uma fila que não sabe pra onde vai te levar* […] *pra conseguir medicações.* (P11, travesti, 33 anos)

A voz da depoente amplia a compreensão ao situar as barreiras no descompasso entre políticas públicas e a realidade do cuidado. Embora exista formalmente o direito à saúde, este se mantém apenas no plano normativo:

[…] *o acesso à saúde,* […] *está no papel, mas não é executado. Tem muitas pessoas trans, travestis do interior. Sou do interior de Minas; lá não tem uma política para pessoas trans. Vim conhecer o ambulatório trans aqui* […] *tive que vir pra uma capital pra questões de melhoria de vida. Se tivesse lá, estava me hormonizando sozinha, não tinha acompanhamento psicossocial*. (P10, travesti, 35 anos)

*Tem um tal de clínico geral. O clínico geral não vai suprir minhas necessidades. Preciso de um profissional endocrinologista.* (P6, homem trans, 32 anos)

Algumas narrativas indicam que embora o acesso à saúde por pessoas trans esteja formalmente garantido, o percurso se fragiliza diante de negativas e ausência de serviços integrados, produzindo desigualdade no cuidado:

*Quando me deram várias negativas, desisti de procurar pelo SUS. Estava peregrinando Distrito Federal atrás de um particular.* (P1, homem trans, 35 anos)

*Eles* (UBS) *não respeitam a identidade de gênero; mandam procurar o ambulatório.* (P7, mulher trans, 28 anos)

O eixo 3 revelou práticas que reduziram constrangimentos e favoreceram a continuidade do cuidado. As pessoas participantes relataram vivências nas quais houve escuta atenta, apoio emocional e atendimento acolhedor, especialmente na atenção à saúde mental. Esses aspectos são ilustrados nas falas:

*Psicólogo*, às *vezes fico pensando… esse meu doutor é cabuloso porque conversa*. Às *vezes, ele nem fala nada, só fica me observando. Aí fala alguma coisinha* […] *parece que ele me escutando alivia e traz paz. Nunca me senti assim.* (P3, homem trans, 29 anos)

[…] *fiz acompanhamento psicológico pelo Centro de Referência Especializado em Assistência Social da Diversidade. Fiz e foi maravilhoso o atendimento, muito bem acolhida, bem assistida. Mas procurei o HUB. Também tive tratamento psicológico… sensacional o atendimento*. (P8, mulher trans, 25 anos)

As pessoas participantes relataram situações em que foram atendidas de forma respeitosa, sendo reconhecidas pelo nome social:

*Fui muito bem atendido.* […] *o atendimento é muito bom. Fui respeitado. Chamaram pelo nome social desde o início. Me sinto seguro nesse lugar.* (P1, homem trans, 35 anos)

[…] *maravilhoso! Sabem nos respeitar; perguntam se a gente quer realizar hormonização, porque tem trans que não quer se hormonizar, e aí é feito tudo com muito carinho e amor e respeito, principalmente respeito. Respeitam o nome social principalmente. Me sinto muito acolhida.* (P2, mulher trans, 22 anos)

O suporte em serviços de referência foi mencionado, destacando a importância da continuidade do cuidado e da inclusão da família no processo:

[…] *tive muita ajuda psicológica no centro de referência* […] *faziam de tudo pra te acolher: conversar, saber questões financeiras, se estava tudo certo, se precisava de alguma coisa. Fiz acompanhamento até em casal com a minha esposa. Me encaminharam pro hospital materno infantil… tive um excelente atendimento com a psiquiatra.* (P5, homem trans, 33 anos)

Além da saúde mental, houve boas referências à prática clínica. Um depoente relatou o atendimento ginecológico, enquanto outro destacou a importância da enfermagem:

[…] *achava que seria bem mais pesado na parte de ginecologista,* […] *o médico foi conversando, foi desenvolvendo, então isso foi me deixando mais calmo,* […] *foi bem mais tranquilo do que poderia ter sido.* (P3, homem trans, 29 anos)

[…] *precisei tomar o hormônio. Fui e a moça* (enfermeira) *me atendeu super bem. Falou: “Pode vim aqui, compra a seringa que eu vou aplicar sua medicação”.* […] *na Unidade Básica de Saúde também.* (P5, homem trans, 33 anos)

Este eixo revelou que, mesmo com desafios, existem vivências positivas marcadas por respeito, escuta e práticas clínicas resolutivas. Esses achados, ancorados nas experiências das pessoas participantes, dialogam com a literatura nacional e internacional, como discutido a seguir.

## DISCUSSÃO

Os relatos apresentados no eixo 1 mostraram que a discriminação e a exclusão não se limitam ao plano individual, negando a legitimidade das identidades de gênero. Os participantes relataram falta de respeito à identidade de gênero (P2) e constrangimentos ligados ao uso de espaços sociais e relacionados à saúde (P14). A negação institucional, traduzida em constrangimentos e falta de acolhimento, também foi apontada em outro estudo^(15)^.

Os relatos de P2, P10 e P14 indicam a cisnormatividade presente nos serviços de saúde: identidades questionadas, autonomia negada e uso indevido de mediadores (familiares). Esse padrão não é apenas local. Na prática, isso se expressa quando P10 relata ter sido ignorada pela médica. A cisnormatividade naturaliza a norma binária de gênero e torna inteligíveis determinadas formas de existência. Esse processo cria fronteiras rígidas. Assim, quando identidades trans rompem essa lógica, são colocadas à margem^(2)^. Essa é uma sistemática que fortalece preconceitos e afeta o acesso à saúde.

Em diferentes países, estudos mostram que o preconceito segue moldando a vida de pessoas trans. Em estudos norte-americanos, pessoas trans também enfrentam discriminação e exclusão social que influenciam o acesso à saúde. No Brasil, a literatura registra obstáculos semelhantes no atendimento^(10)^, reforçando que a barreira é estrutural. A realidade observada em Brasília converge com esse cenário, no qual a cisnormatividade permeia a entrada das pessoas transgêneras nos serviços e sua dificuldade de permanência^(1)^.

As narrativas de P10 e P14 mostram não apenas a deslegitimação da identidade, mas também a negação da autonomia. Tais práticas atuam restringindo a legitimidade de determinadas existências e reproduzindo exclusões que repercutem no acesso à saúde. Na Europa, pesquisa com 307 pessoas trans identificou que mais de 40% precisavam convencer os(as) profissionais sobre a legitimidade de suas necessidades de saúde^(4)^. Esse cenário expressa a dificuldade de reconhecimento da diversidade, mas também a busca insistente por cidadania e pelo direito de existir^(7,20)^. A marginalização social e institucional vivenciada por pessoas trans resulta de desigualdades históricas^(14)^, reforçando a urgência de ações intersetoriais para efetivar políticas públicas de equidade^(12)^.

Os relatos do eixo 2 expõem barreiras concretas enfrentadas pelas pessoas trans no acesso aos serviços de saúde, que vão desde a Atenção Primária à Saúde (APS) até os serviços especializados. A experiência de P1 descreve longas trajetórias e várias negativas. Já P11 mostra a demora para conseguir medicação, mostrando a peregrinação em busca de cuidado. Esses achados sugerem obstáculos no reconhecimento e na organização dos fluxos assistenciais, algo que se repete em diferentes contextos do SUS.

A necessidade de deslocamento em busca de cuidado em saúde, destacada por P10, frequentemente leva as pessoas trans a peregrinar em busca de serviços estruturados. Esse movimento reflete uma lógica ampla de exclusão. Na prática, significa refazer trajetórias, gastar tempo e energia para ter direitos mínimos garantidos. Quando os serviços de saúde não respeitam o nome social e o reconhecimento identitário, produzem afastamento e forçam a busca de alternativas fora do SUS^(11)^.

Na Bélgica, Holanda e Alemanha, barreiras estruturais, permeadas pela centralização dos serviços e pelo alto custo com deslocamento, repercutem sobre o cuidado e o bem-estar das pessoas trans^(4)^. O adiamento no cuidado relacionado a questões estruturais e financeiras também foi identificado na realidade norte-americana^(1)^, o que revela que as barreiras de acesso e a exclusão das pessoas trans no âmbito da saúde não se restringem ao Brasil, mas também estão presentes em países desenvolvidos e com maior disponibilidade tecnológica em saúde. P10 associa, ainda, o acesso à saúde à efetivação de políticas e de serviços inclusivos. As políticas, contudo, ainda apresentam lacunas na implementação, sobretudo no tocante ao financiamento, ampliando desigualdades na atenção à saúde à população transgênera^(7,11,20)^.

A morosidade nos agendamentos, como relatado por P11, e os quatro encaminhamentos, descritos por P1, revelam o peso das barreiras logísticas. Esses entraves afetam diretamente o cuidado. A assistência está baseada, com frequência, em ações individuais de alguns profissionais, sem planejamento ou acompanhamento dos desfechos. Em muitos casos, a busca por modificações corporais ocorre em contextos de vulnerabilidade e invisibilidade^(9)^. Esse dado não é irrelevante, pois mostra o quanto a precariedade atravessa escolhas pessoais e corpos.

A insuficiência de especialistas foi destacada por P6: “Preciso de um endocrinologista; clínico geral não resolve minhas necessidades”, mostrando que a formação acadêmica disponível não atende às demandas específicas. Nesse contexto, surgem ainda as questões do preconceito^(25)^ e do estigma social, que envolvem a pessoa trans e impactam negativamente o cuidado em saúde^(7)^. Isso posto, as dimensões política, estrutural e institucional que moldam o acesso à saúde por pessoas trans no SUS demandam revisão crítica e transformações efetivas^(26)^. Na prática, isso significa que algumas pessoas conseguem avançar no SUS, enquanto outras enfrentam maiores desafios, o que resulta em uma exclusão seletiva que decide quais vidas são reconhecidas^(27)^.

Os dados da pesquisa mostram a busca contínua por atendimento, o que reafirma o caráter ativo e resiliente da população trans diante de barreiras que dificultam o acesso e expõem a precariedade dos serviços disponíveis. Ainda que enfrentem barreiras, emergem resistências, mas também desgaste e desistência^(20,25)^. Algumas pessoas participantes relataram alternativas: entre as(os) 19 participantes, ao menos cinco citaram a automedicação como estratégia recorrente e seis relataram a busca por serviços de saúde privados, assim como P1, evidenciando negligência institucional. Essas narrativas podem ser compreendidas como uma forma de agenciamento e resistência, em que pessoas trans reorientam a própria trajetória de cuidado diante da escassez de serviços públicos.

P7 menciona o encaminhamento da APS: “Eles mandam procurar o ambulatório”. Assim, a APS, embora prevista como porta de entrada, ainda não assume plenamente esse papel diante das demandas trans. Essa realidade expressa seletividade do cuidado, com efeitos desiguais sobre quem consegue ou não ser atendido^(27)^. Estudos realizados nos EUA^(28)^, na Espanha^(29)^ e na África^(6)^ mostram que as barreiras estruturais se repetem, confirmando que os desafios da transgeneridade são globais.

A falta de preparo das equipes fragiliza o reconhecimento das necessidades da pessoa transgênera. Esse achado converge com estudos internacionais, que apontam a dificuldade da APS em atuar como porta de entrada efetiva para pessoas trans em diferentes sistemas de saúde^(27)^. O fortalecimento da APS é estratégico para superar barreiras e garantir integralidade do cuidado, a partir da qualificação permanente dos(as) profissionais^(12,16)^. Esse descompasso se mostra presente em Brasília, sede do poder político nacional, onde existe apenas um ambulatório especializado no cuidado à pessoa trans, considerado insuficiente diante da demanda^(13)^. Nesse contexto, a proposta universalista que orienta o SUS encontra inúmeros entraves à efetivação prática^(26)^.

Os achados indicam insuficiências centradas na APS e apontaram uma rede fragmentada, que transfere para os sujeitos a responsabilidade de negociar o acesso ao cuidado. Esse cenário mostra a necessidade de articulação da rede, de modo a reduzir as desigualdades e garantir a continuidade do cuidado^(7,9)^.

Os relatos do eixo 3 trazem uma dimensão de acolhimento, expressa no uso do nome social e na escuta atenta de alguns profissionais. P8 destacou o atendimento psicológico. P3 relatou que “A abordagem da ginecologia me deixou mais à vontade”. E P5 ressaltou: “Fiz acompanhamento até em casal com minha esposa”. Essas narrativas revelam que, apesar das barreiras, existem práticas sensíveis, centradas na escuta, no respeito ao nome social e na adequação à singularidade. Mostram ainda a valorização do contexto familiar e social, o reconhecimento das decisões individuais (hormonização) e a redefinição da experiência de cuidado, reafirmando a importância de estratégias sensíveis que reduzam a exclusão no SUS. Esse achado corrobora estudos que evidenciam o papel dos familiares no fortalecimento da identidade de gênero e na proteção à saúde mental de pessoas trans^(4)^.

O acompanhamento psicológico é essencial para o ­bem-estar da pessoa, como referiu P8, demonstrando a potência de práticas sensíveis mesmo em meio à escassez institucional. Esse dado demonstra que a saúde mental das pessoas trans não está desconectada das condições estruturais que permeiam o acesso à saúde. Nesse contexto, P5 destacou a condução da enfermeira, cuja atuação pôde desempenhar papel estratégico na implementação de práticas inclusivas, como escuta qualificada e acolhimento, em diálogo com as diretrizes de equidade^(30)^. As práticas de humanização fortalecem vínculos e reduzem os impactos da discriminação/exclusão, reafirmando a importância da formação das equipes de saúde^(11,17,19,20)^. Ainda que essas situações sejam pontuais diante do cenário de exclusão, elas sustentam o vínculo de confiança e a continuidade do cuidado, indicando que o SUS pode ser um espaço de segurança e reconhecimento para pessoas transgêneras.

As limitações deste estudo se relacionam ao número de pessoas participantes e à sua realização em serviços específicos do SUS, situados em um contexto urbano delimitado, o que impede a generalização dos achados. Trata-se de análise situada, que expressa experiências e sentidos produzidos em um determinado território e arranjo assistencial, reconhecendo-se que diferentes contextos institucionais e regionais podem produzir experiências distintas de acesso e cuidado às pessoas trans. Ainda assim, os resultados oferecem contribuições relevantes para a compreensão das dinâmicas de acesso e cuidado ao evidenciarem barreiras institucionais, práticas cisnormativas e modos de organização dos serviços que incidem sobre a produção do cuidado. Esses conhecimentos não visam universalização, mas podem subsidiar reflexões críticas e o aprimoramento de fluxos, práticas e linhas de cuidado em contextos semelhantes.

## CONCLUSÃO

Este estudo teve como objetivo analisar as experiências de acesso aos serviços públicos de saúde por pessoas transgêneras na capital do Brasil. Os resultados evidenciam que o acesso, embora pactuado, permanece condicionado por barreiras estruturais e práticas que reproduzem a cisnormatividade institucional. A pesquisa revelou a coexistência de experiências de exclusão e de acolhimento, destacando que, ao lado da invisibilidade e da violência simbólica, também emergiram boas práticas no acesso à saúde, sobretudo no contexto da atenção especializada.

Os relatos mostraram que o cuidado foi vivido como seguro quando pautado pelo respeito ao nome social e pelo reconhecimento da identidade de gênero, evidenciando a centralidade da equidade como princípio do SUS. A descentralização de serviços especializados, mediante fluxos entre níveis de atenção, a qualificação do acolhimento e a garantia de formação permanente intersetorial são estratégias para reduzir as barreiras no cuidado à saúde. Esses são alguns caminhos para a conformação de um cuidado verdadeiramente emancipatório para o coletivo trans.

## Data Availability

Todo o conjunto de dados que dá suporte aos resultados deste estudo está disponível mediante solicitação ao autor correspondente.

# Translation: Acceso a los servicios de salud pública para personas transgénero: experiencias en la capital de Brasil*

**DOI:** 10.1590/1980-220X-REEUSP-2025-0273es

**Authors:** Ester Mascarenhas Oliveira, Carle Porcino, Cleuma Sueli Santos Suto, Hellen Torres Coelho, Paula Bastos Antunes, Dejeane de Oliveira Silva, Paulo Alberto Moura Marques, Luciana Neves da Silva Bampi

**Affiliations:** 1 Universidade de Brasília, Programa de Pós-Graduação em Enfermagem, Brasília, DF, Brasil.; 2 Escola Bahiana de Medicina e Saúde Pública, Salvador, BA, Brasil.; 3 Universidade do Estado da Bahia, Campus VII, Senhor do Bomfim, BA, Brasil.; 4 Centro Universitário de Brasília, Brasília, DF, Brasil.; 5 Universidade Federal de Minas Gerais, Residência em Obstetrícia, Belo Horizonte, MG, Brasil.; 6 Universidade Estadual de Santa Cruz, Ilhéus, BA, Brasil.; 7 Universidade de Brasília, Faculdade de Ciências da Saúde, Programa de Pós-Graduação em Enfermagem, Brasília, DF, Brasil.

**Keywords:** Personas Transgénero, Accesibilidad a los Servicios de Salud, Equidad en el Acceso a los Servicios de Salud, Barreras de Acceso a los Servicios de Salud, Sistema Único de Salud

## Abstract

**Objetivo:**

Analizar las experiencias de acceso a los servicios de salud pública de personas transgénero en la capital de Brasil.

**Método:**

Estudio cualitativo con 19 personas transgénero vinculadas a tres servicios de salud en Brasilia. Los datos recopilados mediante entrevistas en profundidad se analizaron léxicamente utilizando la Clasificación Jerárquica Descendente, con la ayuda del software Interface de R pour les Analyses Multidimensionnelles de Textes et Questionnaires.

**Resultados:**

Surgieron cinco clases temáticas, organizadas en tres ejes: “Experiencias y disputas por el reconocimiento en espacios públicos e institucionales”; “Barreras estructurales y vías de acceso a la salud”; y “Atención a la salud mental y experiencias exitosas en la acogida de pacientes”.

**Conclusión:**

El acceso a la atención médica para las personas transgénero se ve marcado por barreras estructurales, actitudinales y simbólicas que perpetúan la desigualdad y la exclusión en la atención. Las experiencias positivas identificadas sugieren maneras de fortalecer las prácticas inclusivas, reafirmando la salud como un derecho garantizado por la ley brasileña.

## INTRODUCCIÓN

Las personas transgénero o personas trans constituyen un término genérico que abarca a transexuales y travestis, cuya identidad de género no se corresponde con el sexo asignado al nacer. Las personas cisgénero, por su parte, se identifican con el sexo designado al nacer^(1)^. Este contraste pone de manifiesto tensiones profundas, marcadas por la exclusión y la violencia producidas por la cisnormatividad, que niega a las personas trans la plena ciudadanía^(2)^. Considerar estas dimensiones es fundamental para comprender las barreras que afectan el acceso^(3)^ y los flujos asistenciales en el Sistema Único de Salud (SUS).

Estudios internacionales demuestran la existencia de barreras comunes en el acceso a la salud por parte de las personas trans, ya sea en los Estados Unidos de América (EE.UU.)^(4)^ o en Europa^(1)^, que van desde la discriminación y el uso inadecuado de nombres hasta la falta de profesionales capacitados. En países de América Latina^(5)^ y África^(6)^, a esto se suman la falta de políticas públicas y las limitaciones económicas, que impiden a las personas transgénero acceder a los servicios privados de salud.

En Brasil, estas barreras limitan la integralidad de la atención^(3,7,8,9)^, contribuyen a la exclusión social y a la invisibilidad de las demandas de las personas transgénero en el SUS^(10,11)^, y ponen de manifiesto la incipiente naturaleza de la Política Nacional de Salud Integral de Lesbianas, Gays, Bisexuales, Travestis y Transexuales (PNSI-LGBT). Instituida en 2011, la PNSI-LGBT^(12)^, aunque representa un hito normativo, carece desde entonces de procesos sistemáticos de revisión o evaluación, lo que contribuye a las dificultades observadas en su implementación. En el día a día de los servicios, estas dificultades se manifiestan a través de la persistencia de prácticas cisnormativas y la discontinuidad de los flujos asistenciales, lo que repercute directamente en el proceso de salud-enfermedad de las personas transgénero. Así, el entorno de atención se convierte en un “no lugar”, lo que refuerza la invisibilidad de este colectivo en lo que respecta al acceso^(10,13)^, con consecuencias directas sobre la salud de estas personas.

Las situaciones de negligencia institucional, sumadas a un escenario de violación de derechos, ponen de manifiesto los límites de la protección garantizada por las políticas públicas^(14)^, perpetuando desigualdades, prejuicios y estigmas^(11,15)^, y debilitando la concreción de los principios del SUS^(3)^. La creación de consultorios especializados, como en el Distrito Federal (DF) y en Pernambuco, representó un avance en la consolidación de la política^(13,16)^. Sin embargo, la existencia de estos servicios no ha asegurado el acceso efectivo a la atención, ante barreras institucionales persistentes^(17)^, lo que mantiene fragilidades en el ejercicio del derecho a la salud^(18,19,20)^.

En este contexto, marcado por avances y retrocesos en las políticas, Brasilia, capital de Brasil, es un campo estratégico para comprender las tensiones entre la norma y la práctica. La producción de conocimientos sobre la salud de las personas transgénero en Brasil es limitada y está atravesada por desigualdades regionales. En el DF, los pocos estudios existentes se han dedicado a comprender la actuación de los movimientos sociales y la implementación de la clínica trans^(13)^. Ante este escenario y con el objetivo de contribuir a prácticas más equitativas en el SUS, este estudio busca responder a la siguiente pregunta: ¿cómo experimentan las personas transgénero el acceso a los servicios públicos de salud en Brasilia, capital de Brasil? Este estudio busca analizar las experiencias de acceso a los servicios públicos de salud de las personas transgénero en la capital de Brasil.

## MÉTODO

### Tipo de Estudio

Se trata de un estudio cualitativo y exploratorio, que analiza las experiencias de acceso a los servicios públicos de salud de las personas transgénero en la capital de Brasil. El enfoque cualitativo permite captar significados y trayectorias a partir de las narrativas de las personas participantes, lo que permite sistematizar el fenómeno estudiado^(21)^. Para garantizar la consistencia metodológica y la transparencia del estudio, se observaron los criterios establecidos en la lista de verificación *COnsolidated criteria for REporting Qualitative research* ^(22)^.

### LUGAR

El estudio se llevó a cabo en Brasilia, capital de Brasil, en servicios públicos de salud que actúan como puerta de entrada para la atención de personas trans, incluyendo una Unidad Básica de Salud (UBS), reconocida por su relevancia en la atención de este colectivo debido a su ubicación estratégica, la clínica trans de la Secretaría de Estado de Salud y la Clínica de Salud Mental del Hospital Universitario de Brasilia (HUB). Estos servicios se presentaron como puntos de referencia asistenciales. Aunque son esenciales, operan de manera aislada, sin integración en las líneas de atención.

### Población, Criterios de Selección y Definición de la Muestra

El número de participantes se definió durante el proceso de recolección de datos, considerando el perfil, el acceso a las personas y la saturación de la muestra. Se utilizó la técnica de muestreo *snowball*, adecuada para la temática y el difícil acceso al público investigado. Al final de cada entrevista, los participantes indicaron nuevos contactos. La recolección de datos finalizó en la sexta ronda, debido a la saturación temática^(21)^.

Esta técnica se adoptó porque permite incluir a informantes fuera de los flujos formales de atención, ampliando el alcance de la investigación y reduciendo los sesgos institucionales de selección. Además, la técnica se utilizó porque facilita el acceso y por la sensibilidad ética que implican las investigaciones con la población transgénero.

Al final del proceso, se invitó a 25 personas a participar en la investigación. De ellas, 19 aceptaron, cumpliendo con los siguientes criterios de inclusión: autodefinirse como persona transgénero; tener 18 años o más; y tener experiencia actual o previa en el uso de algún servicio de salud del SUS mencionado en este trabajo. Se definió como criterio de exclusión la presencia de una condición clínica, cognitiva o emocional que impidiera la participación en la entrevista. No se excluyó a ninguna persona de la investigación, pero seis optaron por no formar parte del estudio.

### Recolección de Datos

Esta etapa se llevó a cabo entre marzo y junio de 2022, con la aplicación de un cuestionario sociodemográfico y la realización de entrevistas en profundidad, grabadas y transcritas por la primera autora. Las entrevistas, con una duración media de 50 minutos, se realizaron en un entorno privado. El guion de la entrevista, compuesto por 19 preguntas, fue elaborado por la primera autora y revisado por las tutoras, con el fin de garantizar la claridad y la consistencia metodológica. No se realizó ninguna prueba piloto. No hubo contacto previo entre la investigadora y los entrevistados, lo que redujo posibles sesgos de relación.

Los datos fueron recopilados por la investigadora responsable, una mujer cisgénero, enfermera de salud pública y doctoranda en enfermería con experiencia en investigación cualitativa. La posición de la investigadora en el campo contribuyó a la realización de las entrevistas, favoreciendo la escucha atenta y la profundización de las narrativas.

Se dejó claro que no se realizaría ningún juicio de valor durante las entrevistas. La investigadora tomó notas reflexivas después de las entrevistas, que se utilizaron como apoyo para el seguimiento del proceso de recolección de datos, sin formar parte del corpus analítico del estudio. Se informó a los participantes de que la recolección de datos estaba relacionada con una investigación académica, no vinculada a la atención prestada en los servicios de salud^(22)^.

### Análisis y Tratamiento de los Datos

Los datos demográficos recopilados se transcribieron y se trataron de forma descriptiva. Para ello, se utilizó una hoja de cálculo de *Microsoft Excel*, obteniéndose la frecuencia absoluta y relativa*,* lo que permitió trazar el perfil de las personas investigadas.

Las entrevistas se transcribieron íntegramente en formato digital, se organizaron en un *corpus* textual único y se procesaron mediante el *software Interface de R pour les Analyses Multidimensionnelles de Textes et Questionnaires* ^(23)^. El corpus se sometió a la Clasificación Jerárquica Descendente (CJD), que agrupa los segmentos textuales a partir de la frecuencia y la coocurrencia de términos, generando clases léxicas estadísticamente significativas. Se calcularon índices de aprovechamiento del corpus y porcentajes de retención de las unidades de contexto elementales (UCEs), lo que aseguró la solidez del análisis. La interpretación de las clases siguió los preceptos de Creswell^(24)^, a partir de etapas que incluyeron relectura, codificación, construcción de categorías y articulación entre hallazgos empíricos y literatura.

Este recorrido metodológico se articuló con un marco crítico de género, sexualidad y necropolítica, en diálogo con producciones de autoras y autores trans, buscando poner en tensión las disputas de inteligibilidad que marcan la experiencia de las personas trans en el SUS.

### Aspectos Éticos

El proyecto de investigación fue aprobado por los Comités de Ética en Investigación del Centro Universitario de Brasilia, mediante el Dictamen n.º 5.292.617, y de la Fundación de Enseñanza e Investigación en Ciencias de la Salud del Distrito Federal, mediante el Dictamen n.º 5.419.246. Tras la firma del Consentimiento Libre e Informado, se llevó a cabo la recopilación de datos. Para preservar el anonimato, se identificó a las y los participantes con la abreviatura de la palabra “participante” (P) seguida de un número arábigo. El análisis permitió identificar ejes temáticos, cuyos resultados se presentan a continuación.

## RESULTADOS

La muestra estuvo compuesta por 19 personas transgénero, de las cuales siete eran mujeres trans, dos travestis y diez hombres trans, de entre 18 y 40 años, con una edad promedio de 27 años. La mayoría (n = 10) había completado la educación superior, y dos poseían un título de maestría. De ellos/ellas, 12 declararon tener un ingreso mensual de hasta dos salarios mínimos.

El análisis del corpus dio como resultado 19 textos, 1.511 segmentos de texto, 3.412 formas distintas y una frecuencia media ≥ 12 por forma. La CJD abarcó el 75,58 % del corpus. Surgieron cinco clases, las cuales evidenciaron dimensiones del acceso a la atención de salud por parte de personas transgénero. La clase 1 concentró el 23,4 % del contenido, mientras que las demás presentaron una distribución relativamente homogénea en cuanto a la cantidad de UCE. Los discursos se analizaron en tres ejes temáticos: eje 1 (clases 1 y 4) —Experiencias y disputas por el reconocimiento en los espacios públicos e institucionales; eje 2 (clases 3 y 2): barreras estructurales e itinerarios para el acceso a la salud; y eje 3 (clase 5): atención de salud mental y experiencias exitosas de acogida (Figura 1).

**Figura 1:** Dendrograma de la clasificación jerárquica descendente de entrevistas sobre el acceso a los servicios de salud por parte de personas transgénero en la capital de Brasil. Brasilia, Distrito Federal, 2022 (n = 19).

En el eje 1, surgieron experiencias relacionadas con el reconocimiento de la identidad de género como condición para acceder a la salud. Las narrativas muestran que la falta de validación de las identidades trans en los servicios genera incomodidad y limita el derecho a la salud. Esto se expresa en la forma en que los profesionales tratan a la usuaria o al usuario, como ilustra una participante:

*No puedo ser hipócrita; no saben respetar la identidad de género.* (P2, mujer trans, 22 años)

Además de la interacción con los profesionales, la discriminación y la exclusión también se manifiestan en espacios colectivos, como los servicios de salud y los baños públicos. Estos espacios deberían garantizar la dignidad y el acceso, pero terminan reforzando la cisnormatividad a través de comentarios como:

*La doctora no me miraba; le preguntaba todo a mi mamá.* (P10, travesti, 35 años)

*Me sentí muy mal por unos incidentes que ocurrieron en relación con el uso del baño.* (P14, hombre trans, 23 años)

Estas narrativas indican que las barreras al acceso a la salud para las personas transgénero no se limitan a la falta de servicios, sino que se expresan desde el no reconocimiento de las identidades de género hasta la brecha entre las políticas establecidas y su implementación efectiva.

El eje 2 pone de manifiesto las dificultades cotidianas en el servicio, marcadas por la lentitud en las citas, sucesivas reprogramaciones y la falta de procesos resolutivos. Las personas transgénero informan que recurren a diferentes puntos de entrada, sin recibir respuestas efectivas a sus demandas. Esta situación se expresa en el discurso de la persona participante:

[…] *antes fui al centro de salud, y el centro me derivó al Hospital de Base* […] *tuve tres negativas de los endocrinólogos, lo intenté tres veces* […] *el centro me derivó al HMIB* (Hospital Materno-Infantil de Brasilia) […] *ella me dijo que fuera al HUB* […]. (P1, hombre trans, 37 años)

Existe frustración por la espera y la desvalorización de las necesidades específicas:

[…] *básicamente se tarda muchos meses o incluso años* […] *es una fila que no sabes adónde te va a llevar* […] *para conseguir medicamentos.* (P11, travesti, 33 años)

El testimonio de la entrevistada amplía la comprensión al situar las barreras en el desajuste entre las políticas públicas y la realidad de la atención. Aunque formalmente existe el derecho a la salud, este se mantiene solo en el plano normativo:

[…] *el acceso a la salud,* […] *está en el papel, pero no se aplica. Hay muchas personas trans, travestis del interior. Soy del interior de Minas; allí no hay una política para personas trans. Vine a conocer la clínica trans aquí* […] *tuve que venir a una capital para mejorar mi calidad de vida. Si hubiera existido allí, me estaría administrando hormonas por mi cuenta, no tendría acompañamiento psicosocial*. (P10, travesti, 35 años)

*Hay un tal médico general. El médico general no va a satisfacer mis necesidades. Necesito un endocrinólogo profesional.* (P6, hombre trans, 32 años)

Algunas narrativas indican que, aunque el acceso a la salud para las personas trans está formalmente garantizado, el proceso se debilita ante las negativas y la ausencia de servicios integrados, lo que genera desigualdad en la atención:

*Cuando me dieron varias negativas, dejé de buscar en el SUS.*

*Estaba recorriendo el Distrito Federal en busca de un médico privado.* (P1, hombre trans, 35 años)

*Ellos* (UBS) *no respetan la identidad de género; nos mandan a buscar la clínica.* (P7, mujer trans, 28 años)

El eje 3 reveló prácticas que redujeron las situaciones de incomodidad y favorecieron la continuidad de la atención. Los participantes relataron experiencias en las que hubo escucha atenta, apoyo emocional y atención acogedora, especialmente en la atención de salud mental. Estos aspectos se ilustran en los siguientes comentarios:

*Psicólogo, a veces me quedo pensando… este doctor mío es genial porque conversa. A veces, ni siquiera dice nada, solo se queda observándome. Luego dice alguna cosita* […] *parece que el hecho de que me escuche me alivia y me da paz. Nunca me había sentido así.* (P3, hombre trans, 29 años)

[…] *recibí acompañamiento psicológico en el Centro de Referencia Especializado en Asistencia Social de la Diversidad. Lo hice y la atención fue maravillosa, me sentí muy bien recibida, bien atendida. Pero busqué el HUB. También recibí tratamiento psicológico… la atención fue sensacional*. (P8, mujer trans, 25 años)

Los participantes relataron situaciones en las que fueron atendidos de manera respetuosa, siendo reconocidos por su nombre social:

*Me atendieron muy bien.* […] *la atención es muy buena. Me respetaron. Me llamaron por mi nombre social desde el principio. Me siento seguro en este lugar.* (P1, hombre trans, 35 años)

[…] ¡*maravilloso! Saben respetarnos; nos preguntan si queremos seguir un tratamiento hormonal, porque hay personas trans que no quieren hacerlo, y todo se hace con mucho cariño, amor y respeto, sobre todo respeto. Respetan el nombre social sobre todo. Me siento muy acogida.* (P2, mujer trans, 22 años)

Se mencionó el apoyo en los servicios de referencia, destacando la importancia de la continuidad de la atención y de la inclusión de la familia en el proceso:

[…] *recibí mucha ayuda psicológica en el centro de referencia* […] *hacían de todo para acogerte: conversar, informarse sobre cuestiones financieras, si todo estaba bien, si necesitabas algo. Me hicieron un seguimiento incluso en pareja con mi esposa. Me derivaron al hospital materno-infantil… tuve una excelente atención con la psiquiatra.* (P5, hombre trans, 33 años)

Además de la salud mental, hubo buenas referencias a la práctica clínica. Un entrevistado relató la atención ginecológica, mientras que otro destacó la importancia de la enfermería:

[…]*pensaba que sería mucho más pesado en la parte de la ginecología,* […]*el médico fue conversando, fue explicando, así que eso me fue tranquilizando,* […]*fue mucho más tranquilo de lo que podría haber sido.* (P3, hombre trans, 29 años)

[…]*necesitaba tomar la hormona. Fui y la chica* (enfermera) *me atendió muy bien. Me dijo: “Ven aquí, compra la jeringa y te voy a aplicar la medicación”.* […]*en el Centro de Salud Básico también.* (P5, hombre trans, 33 años)

Este eje reveló que, a pesar de los desafíos, existen experiencias positivas marcadas por el respeto, la escucha y prácticas clínicas resolutivas. Estos hallazgos, basados en las experiencias de las personas participantes, dialogan con la literatura nacional e internacional, como se discute a continuación.

## DISCUSIÓN

Los relatos presentados en el eje 1 mostraron que la discriminación y la exclusión no se limitan al plano individual, negando la legitimidad de las identidades de género. Los participantes informaron de una falta de respeto hacia la identidad de género (P2) y de situaciones de incomodidad relacionadas con el uso de espacios sociales y de salud (P14). La negación institucional, que se traduce en situaciones de incomodidad y falta de acogida, también se señaló en otro estudio^(15)^.

Los relatos de P2, P10 y P14 indican la cisnormatividad presente en los servicios de salud: identidades cuestionadas, autonomía negada y uso indebido de mediadores (familiares). Este patrón no es solo local. En la práctica, esto se expresa cuando P10 relata haber sido ignorada por la médica. La cisnormatividad naturaliza la norma binaria de género y hace inteligibles determinadas formas de existencia. Este proceso crea fronteras rígidas. Así, cuando las identidades trans rompen esta lógica, son marginadas^(2)^. Se trata de un sistema que refuerza los prejuicios y afecta el acceso a la salud.

En diferentes países, los estudios muestran que el prejuicio sigue moldeando la vida de las personas trans. En estudios estadounidenses, las personas trans también enfrentan discriminación y exclusión social que influyen en el acceso a la salud. En Brasil, la literatura registra obstáculos similares en la atención^(10)^, lo que refuerza que la barrera es estructural. La realidad observada en Brasilia converge con este escenario, en el que la cisnormatividad impregna el acceso de las personas transgénero a los servicios y su dificultad para permanecer en ellos^(1)^.

Las narrativas de P10 y P14 muestran no solo la deslegitimación de la identidad, sino también la negación de la autonomía. Tales prácticas actúan restringiendo la legitimidad de determinadas existencias y reproduciendo exclusiones que repercuten en el acceso a la salud. En Europa, una investigación con 307 personas trans identificó que más del 40 % necesitaba convencer a los profesionales sobre la legitimidad de sus necesidades de salud^(4)^. Este escenario expresa la dificultad de reconocer la diversidad, pero también la búsqueda insistente de ciudadanía y del derecho a existir^(7,20)^. La marginación social e institucional que viven las personas trans es resultado de desigualdades históricas^(14)^, lo que refuerza la urgencia de acciones intersectoriales para implementar políticas públicas de equidad^(12)^.

Los relatos del eje 2 exponen barreras concretas que enfrentan las personas trans en el acceso a los servicios de salud, que van desde la Atención Primaria de Salud (APS) hasta los servicios especializados. La experiencia de P1 describe largos procesos y varias negativas. Por su parte, P11 muestra la demora para conseguir medicación, lo que pone de manifiesto la peregrinación en busca de atención. Estos hallazgos sugieren obstáculos en el reconocimiento y la organización de los flujos asistenciales, algo que se repite en diferentes contextos del SUS.

La necesidad de desplazarse en busca de atención de salud, destacada por P10, a menudo lleva a las personas trans a peregrinar en busca de servicios estructurados. Este movimiento refleja una lógica amplia de exclusión. En la práctica, significa rehacer trayectorias, gastar tiempo y energía para que se garanticen derechos mínimos. Cuando los servicios de salud no respetan el nombre social y el reconocimiento de la identidad, generan distanciamiento y obligan a buscar alternativas fuera del SUS^(11)^.

En Bélgica, los Países Bajos y Alemania, las barreras estructurales, marcadas por la centralización de los servicios y el alto costo del desplazamiento, repercuten en la atención y el bienestar de las personas trans^(4)^. El aplazamiento de la atención relacionado con cuestiones estructurales y financieras también se identificó en la realidad estadounidense^(1)^, lo que revela que las barreras de acceso y la exclusión de las personas trans en el ámbito de la salud no se limitan a Brasil, sino que también están presentes en países desarrollados y con mayor disponibilidad tecnológica en salud. P10 asocia, además, el acceso a la salud con la implementación de políticas y servicios inclusivos. Sin embargo, las políticas aún presentan lagunas en su implementación, sobre todo en lo que respecta a la financiación, lo que amplía las desigualdades en la atención de salud a la población transgénero^(7,11,20)^.

La lentitud en las citas, como lo relata P11, y las cuatro derivaciones, descritas por P1, revelan el peso de las barreras logísticas. Estos obstáculos afectan directamente a la atención. La asistencia se basa, con frecuencia, en acciones individuales de algunos profesionales, sin planificación ni seguimiento de los resultados. En muchos casos, la búsqueda de modificaciones corporales se produce en contextos de vulnerabilidad e invisibilidad^(9)^. Este dato no es irrelevante, ya que muestra hasta qué punto la precariedad atraviesa las elecciones personales y los cuerpos.

La insuficiencia de especialistas fue destacada por P6: “Necesito un endocrinólogo; el médico general no resuelve mis necesidades”, lo que muestra que la formación académica disponible no atiende las demandas específicas. En este contexto, surgen además las cuestiones del prejuicio^(25)^ y del estigma social, que afectan a la persona trans y tienen un impacto negativo en la atención de salud^(7)^. Dicho esto, las dimensiones políticas, estructurales e institucionales que moldean el acceso a la salud de las personas trans en el SUS exigen una revisión crítica y transformaciones efectivas^(26)^. En la práctica, esto significa que algunas personas logran avanzar en el SUS, mientras que otras enfrentan mayores desafíos, lo que resulta en una exclusión selectiva que decide qué vidas son reconocidas^(27)^.

Los datos de la investigación muestran la búsqueda continua de atención, lo que reafirma el carácter activo y resiliente de la población trans frente a las barreras que dificultan el acceso y exponen la precariedad de los servicios disponibles. Aunque enfrentan barreras, surgen resistencias, pero también desgaste y desistimiento^(20,25)^. Algunas personas participantes relataron alternativas: entre los 19 participantes, al menos cinco mencionaron la automedicación como estrategia recurrente y seis relataron la búsqueda de servicios de salud privados, al igual que P1, lo que evidencia la negligencia institucional. Estas narrativas pueden entenderse como una forma de agencia y resistencia, en la que las personas trans reorientan su propia trayectoria de atención ante la escasez de servicios públicos.

P7 menciona la derivación de la APS: “Nos mandan a buscar la clínica”. Así, la APS, aunque prevista como puerta de entrada, aún no asume plenamente ese papel ante las demandas trans. Esta realidad expresa la selectividad de la atención, con efectos desiguales sobre quienes logran o no ser atendidos^(27)^. Estudios realizados en EE. UU.^(28)^, en España^(29)^ y en África^(6)^ muestran que las barreras estructurales se repiten, lo que confirma que los desafíos de la transgénero son globales.

La falta de preparación de los equipos debilita el reconocimiento de las necesidades de las personas transgénero. Este hallazgo concuerda con estudios internacionales, que señalan la dificultad de la APS para actuar como puerta de entrada efectiva para las personas trans en diferentes sistemas de salud^(27)^. El fortalecimiento de la APS es estratégico para superar barreras y garantizar la integralidad de la atención, a partir de la capacitación continua de los profesionales^(12,16)^. Este desajuste se observa en Brasilia, sede del poder político nacional, donde solo existe un consultorio especializado en la atención a personas trans, considerado insuficiente ante la demanda^(13)^. En este contexto, la propuesta universalista que orienta al SUS encuentra innumerables obstáculos para su aplicación práctica^(26)^.

Los hallazgos indican deficiencias centradas en la APS y señalaron una red fragmentada, que transfiere a las personas la responsabilidad de negociar el acceso a la atención. Este escenario muestra la necesidad de articular la red, a fin de reducir las desigualdades y garantizar la continuidad de la atención^(7,9)^.

Los relatos del eje 3 aportan una dimensión de acogida, expresada en el uso del nombre social y en la escucha atenta de algunos profesionales. P8 destacó la atención psicológica. P3 relató que “El enfoque de la ginecología me hizo sentir más a gusto”. Y P5 destacó: “Hice el seguimiento incluso en pareja con mi esposa”. Estas narrativas revelan que, a pesar de las barreras, existen prácticas sensibles, centradas en la escucha, el respeto al nombre social y la adaptación a la singularidad. Muestran además la valoración del contexto familiar y social, el reconocimiento de las decisiones individuales (tratamiento hormonal) y la redefinición de la experiencia de atención, reafirmando la importancia de estrategias sensibles que reduzcan la exclusión en el SUS. Este hallazgo corrobora estudios que evidencian el papel de los familiares en el fortalecimiento de la identidad de género y en la protección de la salud mental de las personas trans^(4)^.

El acompañamiento psicológico es esencial para el bienestar de la persona, como señaló P8, demostrando el potencial de las prácticas sensibles incluso en medio de la escasez institucional. Este dato demuestra que la salud mental de las personas trans no está desconectada de las condiciones estructurales que permean el acceso a la salud. En este contexto, P5 destacó la actuación de la enfermera, cuya intervención pudo desempeñar un papel estratégico en la implementación de prácticas inclusivas, como la escucha cualificada y la acogida, en diálogo con las directrices de equidad^(30)^. Las prácticas de humanización fortalecen los vínculos y reducen los impactos de la discriminación/exclusión, reafirmando la importancia de la formación de los equipos de salud^(11,17,19,20)^. Aunque estas situaciones sean puntuales ante el escenario de exclusión, sustentan el vínculo de confianza y la continuidad de la atención, lo que indica que el SUS puede ser un espacio de seguridad y reconocimiento para las personas transgénero.

Las limitaciones de este estudio se relacionan con el número de participantes y con su realización en servicios específicos del SUS, situados en un contexto urbano delimitado, lo que impide la generalización de los hallazgos. Se trata de un análisis situado, que expresa experiencias y significados producidos en un territorio y un sistema de atención determinados, reconociendo que diferentes contextos institucionales y regionales pueden producir experiencias distintas de acceso y atención a las personas trans. Aun así, los resultados ofrecen contribuciones relevantes para la comprensión de las dinámicas de acceso y atención al poner de manifiesto las barreras institucionales, las prácticas cisnormativas y los modos de organización de los servicios que inciden en la prestación de la atención. Estos conocimientos no pretenden la universalización, pero pueden subsidiar reflexiones críticas y el mejoramiento de los flujos, las prácticas y las líneas de atención en contextos similares.

## CONCLUSIÓN

El objetivo de este estudio fue analizar las experiencias de acceso a los servicios públicos de salud de las personas transgénero en la capital de Brasil. Los resultados evidencian que el acceso, aunque acordado, sigue estando condicionado por barreras estructurales y prácticas que reproducen la cisnormatividad institucional. La investigación reveló la coexistencia de experiencias de exclusión y de acogida, destacando que, junto a la invisibilidad y la violencia simbólica, también surgieron buenas prácticas en el acceso a la salud, sobre todo en el contexto de la atención especializada.

Los relatos mostraron que la atención se vivió como segura cuando se regía por el respeto al nombre social y el reconocimiento de la identidad de género, lo que pone de manifiesto la centralidad de la equidad como principio del SUS. La descentralización de los servicios especializados, mediante flujos entre los niveles de atención, la cualificación de la acogida y la garantía de formación permanente intersectorial son estrategias para reducir las barreras en la atención de salud. Estas son algunas vías para la conformación de una atención verdaderamente emancipatoria para el colectivo trans.

## Data Availability

El conjunto de datos que respaldan las conclusiones de este estudio está disponible previa solicitud al autor correspondiente.
